# Evaluation of CRM homogeneity in cases of insufficient method repeatability: Comparison of Bayesian analysis with substitutes for ANOVA based estimates

**DOI:** 10.1016/j.acax.2020.100049

**Published:** 2020-04-14

**Authors:** Thomas P.J. Linsinger

**Affiliations:** Joint Research Centre, Retieseweg 111, Geel, Belgium

**Keywords:** Certified reference material, CRM, Homogeneity, Uncertainty, Bayesian analysis, ISO Guide 35

## Abstract

Insufficient method repeatability is a problem characterising the evaluation of certified reference materials (CRMs). In investigating the homogeneity studies of 216 certified parameters from 36 CRMs released by the European Commission’s Joint Research Centre (JRC) over the last four years, it was found that in 1/3 of the cases, the method repeatability (*s*_r_) was too high to calculate the standard deviation between units (*s*_bb_) by classical analysis of variance (ANOVA). It was also found that the application of the repeatability requirement stated in the ISO Guide 35:2017 is not feasible since it would require unrealistically low repeatability standard deviations or an impossibly high number of replicates per unit.

Evaluation of the uncertainty of homogeneity (*u*_bb_) as evaluated by ANOVA using both the maximum of *s*_bb_ and 0, the maximum of *s*_bb_ and *u*∗_bb_, the uncertainty hidden by method repeatability, the maximum of *s*_bb_ and *s*_bb_/√n and Bayesian analysis, using both informative and diffuse priors, as well as the standard deviation of the unit means, were compared using simulated homogeneity studies with repeatabilities of 1–8% and *s*_bb_ between 0.2 and 2.8%. It was found that using the maximum of *s*_bb_ and *s*_bb_/√n as an estimate of *u*_bb_ guards against severe underestimation but usually results in a severe overestimation of the between-unit variation. Using the maximum of (*s*_bb_, 0) shows the least average bias but results in a severe underestimation of *u*_bb_ in a high fraction of cases. Using the maximum of (*s*_bb_, *u*∗_bb_) limits, but does not completely eliminate cases of a severe underestimation. Also, it leads to average results biased towards high values. For the range of *s*_bb_ and *s*_r_ investigated, Bayesian analysis performed worse than max (*s*_bb_, *u*∗_bb_) in limiting severe underestimation of *u*_bb_, but limited the average bias towards high results.

A risk-based approach to cases of insufficient method repeatability is proposed where, after evaluating the other contributions to the uncertainty of certified values, the effect of severe over- and underestimation of *u*_bb_ is evaluated, and an appropriate approach is chosen based on this analysis.

## Highlights

• The limit for the repeatability standard deviation specified in ISO Guide 35:2017 is not realistic in many cases.
• Bayesian analysis is not guard against severe underestimation of homogeneity in case of poor method repeatability.
• Approaches that guard against underestimation of homogeneity tend to overestimate homogeneity significantly.

## Graphical abstract

**Figure:**
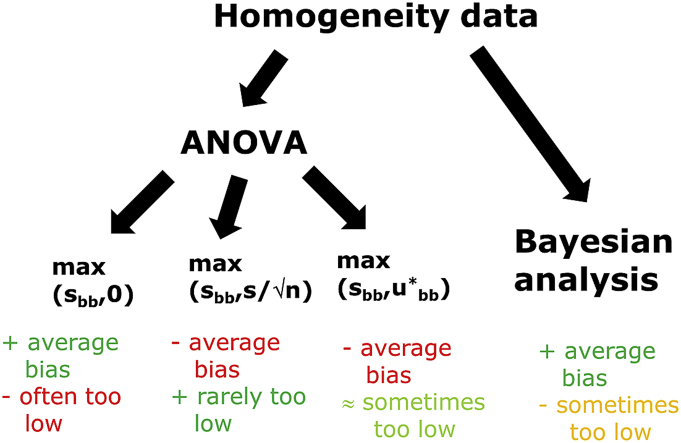

## 1 Introduction

Homogeneity of selected parameters within a batch of material is one of the two defining properties of reference material (RM) [1]. Although ISO Guide 30 does not indicate any specific degree of homogeneity, it, however, states that the degree of homogeneity must be “fit for its intended use in a measurement process”. For certified reference materials (CRMs), ISO 17034 [2] requires the inclusion of an uncertainty contribution from between-unit homogeneity, except when negligible compared to the other uncertainty contributions [3]. This between-unit variation, together with uncertainty contributions from characterisation and stability, leads to the assigned uncertainty of the certified value. Therefore, a quantitative assessment of the variation between different units of a CRM is necessary to either determine the variation directly or to be able to demonstrate that the variation is indeed negligible compared to other uncertainty contributions.

A homogeneity study aims to determine the variation of the certified property between the different units of a CRM batch. A quantitative assessment of homogeneity usually comprises one or more replicate measurements on a representative number of units of a material. The material tested is usually the candidate material itself but can also be a very similar material which can, depending on the validated processing procedures and material properties, serve as a proxy for the homogeneity of the candidate material. The variation of the certified property is a property of the CRM and independent of the chosen measurement method. The variation of the certified property can have different forms like a trend over the whole batch, a few units with outlying values, a sudden change in the certified property due to some change in the manufacturing process etc. However, in many cases, the variation of the average value of each item across the batch can be modelled as Gaussian distribution around the certified value (*μ*_CRM_). In this model, the average properties of the different units *μ*_i_ follow a normal distribution around *μ*_CRM_ with the variance σ^2^_bb_ (Equation (1)).

Equation 1 *μ*_i_ ∼ N (*μ*_CRM_, *σ*^2^_bb_)

The aim of the homogeneity assessment is to estimate the true between-unit variation *σ*^2^_bb_. (Note that ISO Guide 35 [3] refers to the between-unit variation as “between-bottle variation”, thus using the index “bb”. For consistency reasons, this notation is used in this manuscript).

A typical homogeneity study consists of performing n replicate measurements of the certified property on N units of the CRM. Any individual measurement *x*_ij_ on unit i of the CRM will give a result that depends on the true average of the batch (*μ*_CRM_), a potential constant method bias (ε_m_), the deviation of the value of the unit tested from the average of the batch (ε_i_) and a random error reflecting method repeatability (*ε*_ij_) (Equation (2))

Equation 2 *x*_ij_ = *μ*_CRM_ + *ε*_m_ +*ε*_i_ +*ε*_ij_

Usually, only one method is used for all measurements in a homogeneity study. This makes *ε*_m_ constant for the given study. The grand mean of each homogeneity study (*μ*) can, therefore, be written as *μ*_CRM_ + *ε*_m,_ which simplifies Equation (2) to Equation (3).

Equation 3 x_ij_ = *μ* +*ε*_i_ +*ε*_ij_

It is often assumed that the random error *ε*_ij_ is normally distributed with a mean of zero and the repeatability variance *σ*^2^_r_ [3]. In many cases also, *ε*_i_ is assumed to follow a normal distribution with an average of zero and a variation of *σ*^2^_bb_.The variation between units is usually small, so the repeatability variance is the same for all units, making ε_ij_ statistically independent from *ε*_i_. The *i* units tested are regarded as a typical sample of the whole batch and the between-unit effect *ε*_i_ is modelled as a random factor, which means that the basic model, which is given in Equation (3) is a random effects model. In this case, the observed variation of the measurement results (*u*^*2*^_c,bb_) comprises the variation between units (*s*^*2*^_bb_) and the measurement variation (*s*^*2*^_r_) as shown in Equation (4) [4]. In this case, *s*^*2*^_bb_ is an estimate of *σ*^2^_bb_ and *s*^*2*^_r_ is an estimate of *σ*^2^_r_.

Equation 4 $$u_{c,bb}^{2}=s_{bb}^{2}+\frac{s_{r}^{2}}{n}$$

From the data of the homogeneity study, *s*_bb_ is calculated and is then used as estimate of the uncertainty due to between-unit variation (*u*_bb_). It should be noted that a constant method bias is irrelevant, as it effects all measurements in the same way and will therefore not contribute to the observed variation of results.

Traditionally, *s*_bb_ is calculated from the observed variation of all results using analysis of variance (ANOVA) [3] as shown in Equation (5). The basic assumptions of ANOVA are homoscedasticity (homogeneity of variances in each group), normality (values within each group are drawn from a normal distribution) and randomness and independence (the results are random and the units are independent of each other).

Equation 5 $$s_{bb}=\sqrt{\frac{MS_{between}-MS_{within}}{n}}$$

With *MS*_between_ and *MS*_within_ being the mean squared errors between and within groups from ANOVA and *n* the number of replicate measurements performed per unit, *s*_r_ is calculated as √*MS*_within_. Equation (5) is derived from an expression that substitutes the estimates *MS*_between_ and *MS*_within_ for the expected values of these mean squares in the equation that relates the expected variances *σ*^2^_bb_ and σ^2^_r_ with the mean squares. This generally works well as long as *σ*^2^_bb_ is large compared to σ^2^_r_/*n*. For very homogeneous materials (*σ*^2^_bb_ is very small) and/or methods with a high repeatability variance (σ^2^_r_/*n* is large), the argument in the equation can become negative, which makes a calculation of *s*_bb_ impossible. Several solutions have been suggested in this case, either by replacing the estimate for *s*_bb_ from Equation (4) with a non-negative value, by selecting a method with a sufficiently small *σ*^2^_r_ or by performing a sufficiently high number of replicate measurements n per unit that ensures that the argument in the square root does not become negative in the first place or recently by using Bayesian analysis.

### 1.1 Approach 1: choosing a non-negative value

The classic response in frequentist statistics to a negative argument in Equation (5) is to set *s*_bb_ to zero. However, if the measurement method lacked sufficient precision, this can lead to a severe underestimation of the between-unit variation. It has, therefore, been argued that the relation between *s*^*2*^_bb_ and *s*^*2*^_wb_ is of no interest in reference material production [4]. Non-negative estimators for the between-group variance have been proposed by Federer [5]. Alternatively, an attempt has been made to find an approximate estimate for the inhomogeneity that could be hidden by the method repeatability (named *u*∗_bb_). This led to an equation based entirely on the estimated method repeatability and its degrees of freedom. The degrees of freedom, in turn, depend on the total number of units tested *N* and the number of and the number of replicates performed per unit (*n*) [6] (Equation (6)).

Equation 6 $$u_{bb}^{*}=\sqrt{\frac{MS_{within}}{n}\;}\cdot \sqrt[{4}]{\frac{2}{N\left(n-1\right)}}$$

It was suggested to use the larger value of *s*_bb_ and *u*∗_bb_ as an estimate of the uncertainty of homogeneity [6]. This approach is given as an option in the ISO Guide 35 [3] and is followed by several reference material producers. The rationale for the procedure is that if no inhomogeneity is detected, one should rather use the potentially undetected inhomogeneity as a conservative estimate for the undetected inhomogeneity. This approach has recently been criticised not only on fundamental terms but also as being an underestimation of the inhomogeneity that could really be hidden [7]. As an alternative and a more conservative approach, using the larger of *s*_bb_ and *s*_*r*_/√n as an estimate of the between-unit variation has been proposed [8]. In the case of a perfectly homogeneous material (*σ*^2^_bb_ = 0), the variation between unit means will entirely be determined by the random variation *s*_*r*_/√*n*. In the absence of any detected variation, the variation of unit means of perfectly homogeneous material is taken as an estimate of the between-unit variation.

It should be noted that regardless of the value chosen, replacing the nominal value for *s*_bb_ with a non-negative value introduces a bias. The rationale for using these estimates, nevertheless, is that such a positive bias is preferable to the possibility of a severe underestimation of the heterogeneity.

### 1.2 Approach 2: ensuring that the argument does not become negative

ISO Guide 35:2017 [3] and ISO 13528 [9] suggest a different solution to this problem by setting minimum requirements for the repeatability standard deviation (*s*_r_) of the methods used for homogeneity assessment. If these requirements are met, and no variation can be calculated, *s*_bb_ is set at zero. The rationale is the minimum requirements should ensure that *MS*_within_-*MS*_between_ in Equation (5) does not become smaller than zero. If *MS*_within_-*MS*_between_ in Equation (5) becomes smaller than zero nevertheless, then the *s*_bb_ is so small that the very fact of a negative argument in the square root proves that the uncertainty contribution from homogeneity is negligible compared to other uncertainty contributions. To this end, ISO Guide 35 recommends that *s*_r_/√*n* should be smaller than 1/3 of the target uncertainty of the certified value. ISO 13528 has an equivalent criterion, albeit calculated for *n* = 1. This approach is much more established in proficiency testing, where already, the 2005 edition of ISO 13528 stipulated the same criterion but is new for reference materials, where it was first formalised in the 2017 version of ISO Guide 35. The reason might be that the target is easier to reach in proficiency testing than for CRMs: for proficiency tests, the target uncertainty is usually the size of the reproducibility standard deviation of the results submitted by the participants or larger, which makes it relatively easy to find methods that fulfil this criterion.

In contrast, for CRMs, in an ideal case, the uncertainty of the certified value is negligible compared to the measurement uncertainty of the measurement result obtained by the user of the CRM. This leads to much smaller target uncertainties than in proficiency testing. The applicability of this approach for CRMs has, therefore, not yet been tested.

### 1.3 Approach 3: Bayesian analysis

A third and completely different approach to solving the problem of negative arguments in the square root of ANOVA is to abandon the calculation algorithm of the classical ANOVA and use Bayesian analysis instead. It should be emphasised that the basic model remains the same, namely a hierarchical random-effects model. The only difference is in the algorithm in which the model parameters are estimated (prior probability density functions in the Bayesian analysis versus sole reliance on the measured data in the frequentist model). This approach has the advantage that *s*^*2*^_bb_ can be defined as non-negative; hence, eliminating the problem from the onset. The result of Bayesian analyses depends on the choice of priors, therefore, a careful choice is necessary. Van der Veen was the first to apply Bayesian analysis to the evaluation of homogeneity studies reference materials [10]. His analysis of the homogeneity studies of gas mixtures showed the applicability of Bayesian analysis to the evaluation of homogeneity studies. In the examples he investigated, *s*_bb_ tended to be larger than the *u*∗_bb_ calculated according to Ref. [6], and he also suggested that *u*∗_bb_ might not be a good measure for hidden inhomogeneity.

One advantage of using Bayesian analysis is that, unlike the point estimates for *s*_bb_ from ANOVA or approaches 1 and 2, it automatically gives a probability density function of *s*_bb_ which may reflect the poor information about *s*_bb_ better than one number. This could lead to a more realistic assessment of the uncertainty of the certified value, especially in cases where also the uncertainty contributions for characterisation and stability are available as probability density functions. In this case the distribution functions rather than point estimates could be propagates as described in supplement 1 to ISO Guide 98–3 [11] An evaluation of this is, however, beyond the scope of this manuscript.

When assessing the merits of each of these approaches, a balance between two conflicting goals needs to be reached. On the one hand, an ideal approach would never significantly underestimate *u*_bb_, as this would lead to unrealistically small uncertainties of certified values. On the other hand, *u*_bb_ should also not be significantly overestimated to avoid inflating the uncertainties of certified values needlessly. However, the *average* performance of an approach, i.e. whether it consistently over- or underestimates *u*_bb_ is of little or even no importance to the user of a CRM: each user uses only a relatively small number of CRMs. Consequently, a consistent but slight overestimation of the uncertainty is irrelevant compared to on average correct but in some cases, widely deviating results. There is another reason for the irrelevance of small differences: It is recognised that uncertainties are not perfectly known. Therefore, few CRM producers state more than two significant figures for the uncertainties of the certified values. For non-nuclear CRMs produced by the European Commission’s Joint Research Centre (JRC), uncertainties are rounded in a way to ensure that the error introduced by the rounding is between 3 and 30% of the final expanded uncertainties. Slight deviations, therefore, would be covered by the rounding error and are of no consequence to the user. Therefore, individually widely deviating results are of higher importance than the average performance of an approach.

For real materials, as the *σ*_bb_ is unknown, one can only compare whether one approach gives a higher estimate for *s*_bb_ than another. The situation is even more complicated in cases where no homogeneity is detected and where it is not clear whether this is because an approach underestimates the inhomogeneity or whether the other approach overestimates it. Simulated data where an artificially introduced variation between units is known seems an effective way to circumvent this problem: Apart from mere comparison of the results of the different approaches relative to each other, comparison of the estimates with the true values also allows a more solid statement whether an approach over- or underestimates the uncertainty.

In this manuscript, two questions in connection with homogeneity studies of certified reference materials are investigated. The first part uses real data to check whether there is a significant number of homogeneity studies where *MS*_between_ - *MS*_within_ is negative and whether a repeatability limit as proposed by ISO Guide 35 is realistic and implementable. The second part compares different approaches to dealing with cases where *s*_bb_ is equal or smaller than *s*_r_/√n, i.e. cases where it might be impossible to estimate *s*_bb_ from classical ANOVA because the data contain little information on the between-unit variation. To this end, three non-negative replacements for *s*_bb_ and the results of Bayesian analysis with informative and diffuse priors are compared simulated homogeneity studies with known between-unit variations.

## 2 Data and data analysis

### 2.1 Need for an uncertainty estimation for alternative homogeneity evaluations and practicality of a repeatability limit

Homogeneity studies of 36 different certified reference materials comprising 216 certified parameters released by the JRC between 2014 and 2018 were investigated. These materials cover a wide range of matrix/measurand combinations. They include mass fractions of trace elements in chocolate, algae and copper, particle sizes of nano- and microparticles, mass fractions of polycyclic aromatic hydrocarbons and polybrominated flame retardants in water and sediment, enzyme activities of pure enzyme preparations and protein mass fractions in serum. A complete list of the CRMs used, the measurands investigated, and a link to the certification reports containing the data is given in the supplementary electronic materials. Most of these materials are matrix CRMs, and the measurement of the analytes in question usually involves more or less complex sample preparation. Each study consists of two to four determinations of the measurand in question in 10–20 units. 117 of these studies concerned element analysis, 56 small organic molecules, 19 physical properties, 9 operationally defined properties, 11 proteins and 4 measurands based on polymerase chain reaction (PCR). In the first step, 38 studies that could not be evaluated by standard ANOVA because they contained outliers or trends in the filling sequence were excluded. While trends in the filling sequence could be included both in frequentists and Bayesian models, an investigation of such models is beyond the scope of this manuscript. The exclusion of these resulted in 181 studies that could be evaluated by standard ANOVA. The distribution of the selected studies was virtually unchanged by this exclusion: 101 elements, 43 small organic molecules, 13 physical properties, 8 operationally defined properties, 10 proteins and 4 PCR-based measurands.

For each of the studies selected, *s*_bb_ and *s*_r_ as calculated from ANOVA were taken from the certification reports, as were *u*∗_bb_ and *u*_char_. The number of cases where *s*_bb_ could not be calculated as *MS*_between_ was smaller than where *MS*_within_ were counted and the number of replicates that would have to be performed to fulfil the criterion *s*_r_/√n ≤ *u*_char_ with *u*_char_ being the uncertainty of characterisation. Subsequently, these data were summarised as median values to minimise the effect of single extreme values.

### 2.2 Analysis of simulated homogeneity data

#### 2.2.1 Data generation for simulated homogeneity studies

Sets of 20 homogeneity studies comprising 15 units analysed in triplicate were generated. The simulated *σ*_r_ were 1%, 3%, 5% and 8% and the applied *σ*_bb_ were 0.2%, 0.5%, 1% and 2.8%. Sets of 900 normally distributed random numbers with an average of 100 and a standard deviation of 1%, 3%, 5% and 8% were generated using the website www.random.org. These 900 numbers were grouped in 300 sets of three results, each set representing the three replicates of one unit. 300 random numbers with an average of 1 and standard deviations of 0.002, 0.005, 0.010 and 0.028 were generated using the website www.random.org. These random numbers represent the deviation of each unit from the average measurand level of 100. Each of the 900 random numbers of each set was then multiplied by the variation factor to simulate the measurement results on this unit (Equation (7)).

Equation 7 *x*_ij_ = *z*_ij_∗*y*_i_

*x*_i,j_ simulated result
*z*_i,j_ individual random number with i = 1 to 300 (unit number) and j = 1 to 3 (replicate number)
*y*_i_ with i = to 300: fraction of each unit mean of the average of the study
The results from i = 1 to i = 15 are homogeneity study 1, i = 16 to i = 30 are homogeneity study 2 etc.

As seen in Equation (7), a multiplicative bias is applied rather than an additive one (the factor for the between-unit variation is multiplied with the random numbers for the replicates). This was done in line with the observation that for many methods, the relative variations are constant. Due to the relatively small bias (0.2–2.8% between-unit variation), the differences between multiplication and addition are negligible.

**Fig. 1:**
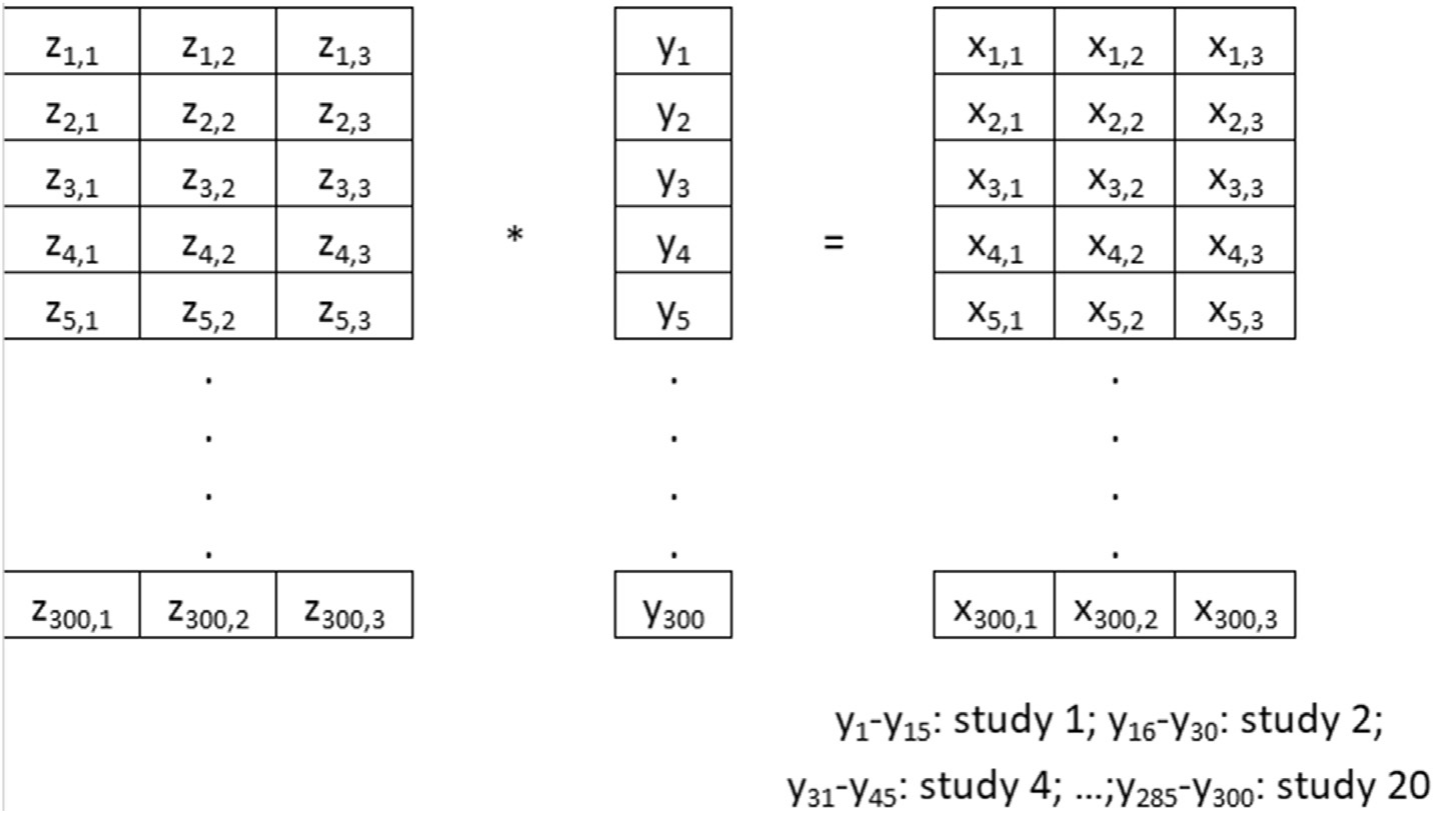
Overview of the data generation of the simulated homogeneity studies.

The complete set of random numbers is given in the supplementary electronic material, and an overview of the calculation is given in Fig. 1. The combination of *s*_r_ and *s*_bb_ used are shown in Table 1.

**Table 1:** Combinations of σ_r_ and σ_bb_ used.

*σ*_r_	σ_bb_	Ratio σ_r_/σ_bb_
1%	0.2%	5
	0.5%	2
	1%	1
	2.8%	0.33
3%	0.2%	15
	0.5%	6
	1%	3
	2.8%	1
5%	0.2%	25
	0.5%	10
	1%	5
	2.8%	1.67
8%	0.2%	40
	0.5%	16
	1%	8
	2.8%	2.67

### 2.2.2 ANOVA and non-negative estimates of *s*_bb_

The simulated data were evaluated using classical one-way ANOVA calculating *s*_r_, *s*_bb_ and *u*∗_bb_. Three approaches to dealing with cases where *MS*_between_ < *MS*_within_ were applied. In one case, *u*^*2*^_bb_ was estimated as the maximum of s_2bb_ and 0, i.e. setting *u*_bb_ equal to zero in case *MS*_between_ < *MS*_within_. The second case was to set *u*_bb_ as the maximum of *s*_bb_ and *u*∗_bb_, i.e. setting a lower limit and the third case was to set *u*_bb_ as the maximum of *s*_bb_ and *s*_r_/√n.

Throughout the manuscript, the following codes are used:

“A-max(*s*_bb_,0)” for evaluation by one-way ANOVA where *s*_bb_ was set to zero for all cases where *MS*_between_ < *MS*_within_.
“A-max(*s*_bb_, *u*∗_bb_)” for evaluation by one-way ANOVA where *s*_bb_ was set to the maximum of *s*_bb_ and *u*∗_bb_ as proposed in Ref. [6].
“A-max(*s*_bb_, *s*_r_/√n)” for evaluation by one-way ANOVA where *s*_bb_ was set to the maximum of *s*_bb_ and *s*_r_/√n as proposed in Ref. [8].

### 2.2.3 Bayesian analysis

#### 2.2.3.1 Definition of the model

Each study was evaluated using WinBUGS 1.4.3 (Imperial College and MRC, UK) involving the hierarchical WinBUGS program given in Table 2. The model itself follows the model described in the introduction:

**Table 2:**
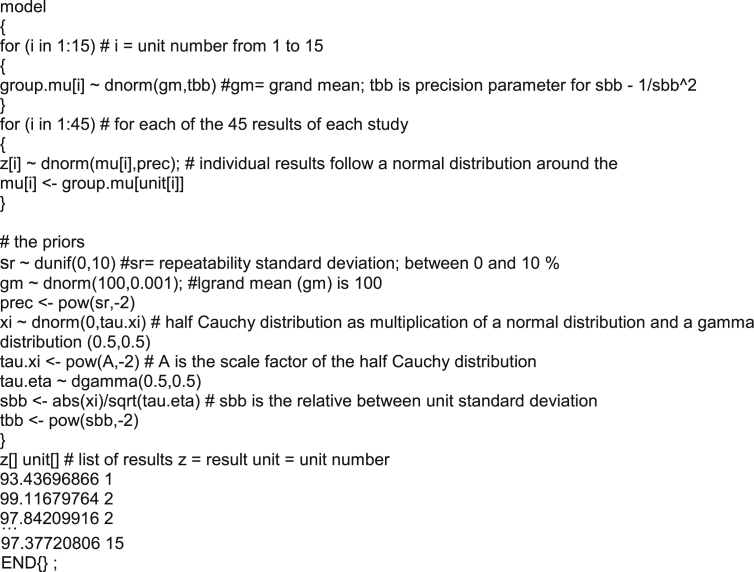
WinBUGS® Program for the evaluation of homogeneity studies.

Each of the units means i (mu[i] in the Winbugs code) follows a normal distribution around the grand mean (gm in the Winbugs code) with a variance *σ*^2^_bb_ (Equation (1)). The individual measurement results (z[i] in the Winbugs code) follow a normal distribution around the respective unit mean with a standard deviation *s*_r_. As Winbugs has no integrated function for the Cauchy distribution, the fact that if x_i_ ∼ N(0, B) and k∼Γ(0.5, 0.5), then x_i_/√k follows a Cauchy distribution with the scale parameter B, which was exploited as was done in Ref. [12].

**Fig. 2:**
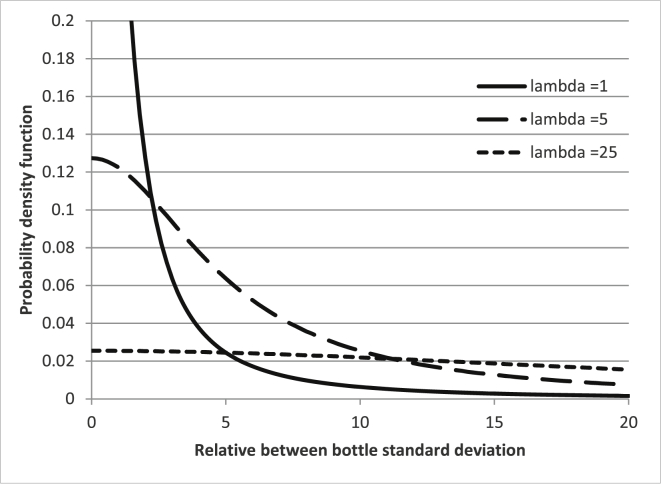
Probability density functions of half-Cauchy distributions with a scale parameter of 1 (solid line) 5 (dashed line) and 25 (dotted line).

The following priors were chosen:

Grand mean (gm in the code): Normal distribution with *μ* = 100 and *σ* = 31. The rationale for this prior is that the data of the studies were chosen such that the grand mean should be 100. The *σ* = 31 of grand mean is in the view of this a rather diffuse prior and was only chosen to ensure that the calculated grand mean is within this range.
unit means i (group.mu[i] in the code): normal distribution with *μ* = grand mean and *σ* = *s*_bb_. This prior directly follows from the model where the unit means are assumed to be normally distributed around the grand mean.
*s*_r_ uniform prior between 1 and 10% for repeatabilities of 1%, 3% and 5% and a uniform prior between 1% and 20% for the tests with a repeatability of 8%. The goal was to have a mildly informative prior for *s*_r_. The choice of this prior reflects real life where one usually has a reasonable idea about the repeatability standard deviation of the method from the method validation study. However, due to changes in instrument performance, etc., the repeatability standard deviation on a single day may be worse, so the limit was increased. The prior itself is of little importance, as sufficient data points are available to provide a good estimate of s_r_.
*s*_bb_: Half Cauchy distributions were used as prior for *s*_bb,_ as suggested in Ref. [12,13] and applied in Ref. [10]. Three variants of this prior were applied. The first one was a diffuse prior with a scale parameter lambda of 25. This prior allows virtually all values for *s*_bb_ even to very high ones. Furthermore, two informative priors were applied. For nominal *s*_bb_ of 0.2%, 0.5% and 1%, a scale parameter of 1 was applied, which severely limits the probability of high *s*_bb_. For nominal *s*_bb_ of 2.8%, a scale parameter of 5 was applied, which means that most values chosen for *s*_bb_ are <5. The relevant parts of the probability density function of half Cauchy distributions with scale parameters of 1, 5 and 25 are shown in Fig. 2.

For each study, 100000 iterations were performed. A thinning of 20 was applied to reduce the autocorrelation in *s*_bb_, resulting in 5000 valid iterations per study. In all cases, the median of the 5000 *s*_bb_ was used as an estimate of *u*_bb_. The choice of the median rather than the average as an estimate for *s*_bb,_ results in slightly lower values, especially in cases where *s*_bb_ is equal or smaller than *s*_r_ because in these cases, skewed distributions for the posterior are obtained. It was found that the difference is about 3 relative % in case *MS*_between_ = *MS*_within_ = 3% and about 20 relative % in the most extreme case where *s*_bb_ = 0.2% and *s*_r_ = 8%.

Throughout the manuscript, the following codes are used:

“B-uninf” for the results of the Bayesian analysis applying a scale parameter of 25 for the half-Cauchy distribution of *s*_bb_
“B-inf” for the results of the Bayesian analysis with an informative prior for *s*_bb_ using scale parameters of 1 (for nominal *s*_bb_ of 0.2%, 0.5% and 1%) and 5 (for nominal *s*_bb_ of 2.8%), respectively.

#### 2.2.3.2 Validation of the model

The basic model was validated by comparing the data for *σ*_bb_ = 2.8 and *σ*_r_ = 1 to the results from classical ANOVA. This is a situation where, for all data used, *s*_bb_ can be evaluated by ANOVA, and the Bayesian analysis should give the same result. For each of the 20 tests, the ratio between the *s*_r_ obtained by Bayesian analysis and by ANOVA was calculated. This ratio was on average, 1.03 reflecting the difference between median and mean.

Similarly, for each of the tests, the ratio between the *s*_bb_ obtained by Bayesian analysis and *s*_bb,real_ was calculated. This ratio was on average, 0.98 for the uninformed prior and 1.01 for the informed prior. This comparison shows that the model and its implementation are an appropriate implementation of the hierarchical model chosen.

#### 2.2.3.3 Investigation of the influence of the priors

The influence of the scale parameter of the half Cauchy distribution for *s*_bb_ is part of the manuscript, as one of the datasets generated is a diffuse prior with a scale parameter of 25 and one is an informative prior with scale parameters of 1 and 5, respectively.

The goal of this manuscript is to investigate cases where the repeatability standard deviation is larger than *s*_bb_. The influence of the other priors was therefore investigated on a dataset with an *s*_bb_ = 2.6 and an *s*_r_ = 5, as well as on a dataset with *s*_bb_ = 1% and *s*_r_ = 5% for standard deviations of the grand mean of 10 and 3.1, including a less informative prior for *s*_r,_ namely a uniform distribution between 0 and 20. The results are shown in Table 3.

**Table 3:** Results of the influence of various priors. SP = scale parameter of the half Cauchy distribution. In both cases the informative priors were used. s_GM_ = standard deviation of the grand mean.

	SP	Prior *s*_GM_	Prior *s*_r_	*s*_bb_	*s*_r_
*s*_bb_ = 2.8 and *s*_r_ = 5	5	31	0–10	3.272	5.961
		10	0–10	3.246	5.971
		3.1	0–10	3.154	5.990
		31	0–20	3.271	5.967
*s*_bb_ = 1 and *s*_r_ = 5	1	31	0–10	6.114	1.243
		10	0–10	6.119	1.213
		3.1	0–10	6.108	1.210
		31	0–20	6.117	1.189

The results show that a less informative prior for *s*_r_ has no influence on the result, neither for *s*_r_ nor for *s*_bb_. This is an expected outcome since the number of individual results is high enough to dominate the posterior. Also, a reduction of the standard deviation of the grand mean does not change the result, as long as the standard deviation, which is chosen is larger than the combination of *s*_bb_ and *s*_r_/√n (= *u*_c,bb_ of Equation (4)): A standard deviation of 10 does not change the results but a standard deviation of 3.1, in combination of an *s*_bb_ of 2.6 and an *s*_r_ of 5 would result in a lower estimate for *s*_bb_.

Based on this, it is concluded that the priors are adequate for the purpose.

### 2.2.4 Comparison of approaches

Real between-unit standard deviation (*s*_bb, real_): For each simulated study, the real between-unit standard deviation of the study, defined as the standard deviation of the 15 y_i_ of the respective study was calculated. This standard deviation was multiplied with 1.018, the correction factor for 15 data points to obtain an unbiased estimate for the standard deviation [14].

For each study, the ratio between the estimated *u*_bb_/*s*_bb,real_ was calculated, and the following parameters were derived:

• *Average estimate*: For each combination of *s*_bb,real_ and *s*_r,_ the average of the 20 ratios *u*_bb_/*s*_bb,real_ was plotted against the ratio *s*_r_/*s*_bb,real_. For a perfect evaluation method, this ratio would be one for all ratios *s*_r_/*s*_bb,real_. In practice, even for a completely unbiased evaluation, this ratio will scatter around 1 due to the effect of *s*_r_ on the estimate. Systematic deviations from unity indicate a bias in the evaluation approach.
• *Maximum overestimation:* For each combination of *s*_bb,real_ and *s*_r,_ the upper single-sided 95% confidence limit was calculated from the 20 ratios *u*_bb_/*s*_bb,real_. This parameter accounts for the fact that users are affected by an unnecessarily large uncertainty of the CRM they purchased. Whether an evaluation performs well on average is of minor importance. The maximum overestimation was plotted against the ratio *s*_bb,real_/*s*_r_.
• *Maximum underestimation*: For each combination of *s*_bb,real_ and *s*_r_ the lower single-sided 95% confidence limit based was calculated from the 20 ratios *u*_bb_/*s*_bb,real_. As nobody will ever introduce a negative between-unit standard deviation, the lower confidence interval was capped at zero, meaning that values < 0 were replaced by 0. The rationale for this parameter is the same as for the maximum overestimation, namely that a user is affected by an underestimation of the uncertainty of a specific CRM bought and not by an average bias. The maximum underestimation was plotted against the ratio *s*_bb,real_/*s*_r_.
• *Fraction of significant underestimations:* For each combination of *s*_bb,real_ and *s*_r,_ the fraction of the studies with *u*_bb_ < 0.7∗*s*_bb,real_ was calculated. Whereas the maximum overestimation and maximum underestimation investigate extreme events, this parameter quantified the frequency where *u*_bb_ is severely (here defined as more than 30%) underestimated.

## 3 Results and discussion

### 3.1 Need for an uncertainty estimation for alternative homogeneity evaluations and practicality of a repeatability limit

The results of the compilation of homogeneity studies are given in Table 4. In 13–46% of the cases, it was impossible to calculate *s*_bb_ as *MS*_between_ < *MS*_within_. For method defined properties and PCR based methods, *s*_bb_ could not be calculated in 13 and 17% of the cases, respectively. For all the other fields, the fraction of studies where *s*_bb_ could not be calculated ranged from 30 to 46%. This is not because of the selection of inferior methods that show high repeatability standard deviations. The median repeatability standard deviation for elements is 3.4% and for small organic molecules, 5.0%, which is close to the optimum that good laboratories can achieve for a larger number of samples. Smaller repeatability standard deviations for elements can be achieved by, e.g. thermal ionisation isotope-dilution mass spectrometry of elements. However, this method is generally not suitable for the determination of the 20–40 samples required for homogeneity studies. The reason for not being able to calculate *s*_bb_ is rather the good homogeneity of the materials, where median s_bb_ ranges from 0.5 to 1.8%. This means that the problem of negative arguments under the square root in ANOVA is real and present in a large fraction of homogeneity studies of matrix materials.

**Table 4:** Summary of homogeneity studies investigated.

Measurand	Total number of studies	Number of studies where *s*_bb_ could not be calculated	Median *s*_r_	Median *s*_bb_	Median *u*∗_bb_	Median *u*_char_	Median n required for the ISO Guide 35 criterion
Element	101	39	3.40	1.00	0.84	2.30	22
Small organic molecule	43	14	5.04	1.80	1.51	4.33	11
Physical properties	13	6	0.31	0.49	0.12	0.57	3
Method defined properties	8	1	0.88	0.58	0.29	0.65	12
Biomolecule	10	3	2.34	0.96	0.62	0.66	40
PCR	4	0	11.57	4.84	2.26	4.64	111
**Sum**	**179**	**63**	**3.28**	**1.11**	**0.90**	**2.71**	**19**

Despite the good repeatabilities of the methods used in the studies, very few of them would have met the criterion for method repeatability stipulated in ISO Guide 35. The median number of replicates to fulfil this requirement for chemical analyses ranges from 11 to 40. Physical properties form an outlier on the low side, as the methods often are exceptionally repeatable and PCR-based measurands form an outlier on the high side, as here, the assigned values often are not based on PCR itself. Consequently, the requirement combines rather small *u*_char_ with the large standard deviations from PCR. Clearly, performing 10 to 40 replicate measurements on each unit in a homogeneity study is not practical and often impossible due to the limitation of sample per unit. Even if the amount of sample per unit was sufficient to perform this number of measurements and also, if one could commit the resources needed for this large number of measurements (several hundred measurements per study), it is easily possible that the criterion would still not be fulfilled as instrument drift, the time required for this number of sample preparations etc. could increase the observed repeatability standard deviation.

These data show that the repeatability criterion stated in ISO Guide 35 is not achievable for a wide range of matrix CRMs, as either unrealistically small repeatability standard deviations would have to be achieved or an impractically high number of replicates would have to be performed.

### 3.2 Comparison of the different evaluation approaches on the simulated homogeneity studies

#### 3.2.1 Average estimate

**Fig. 3:**
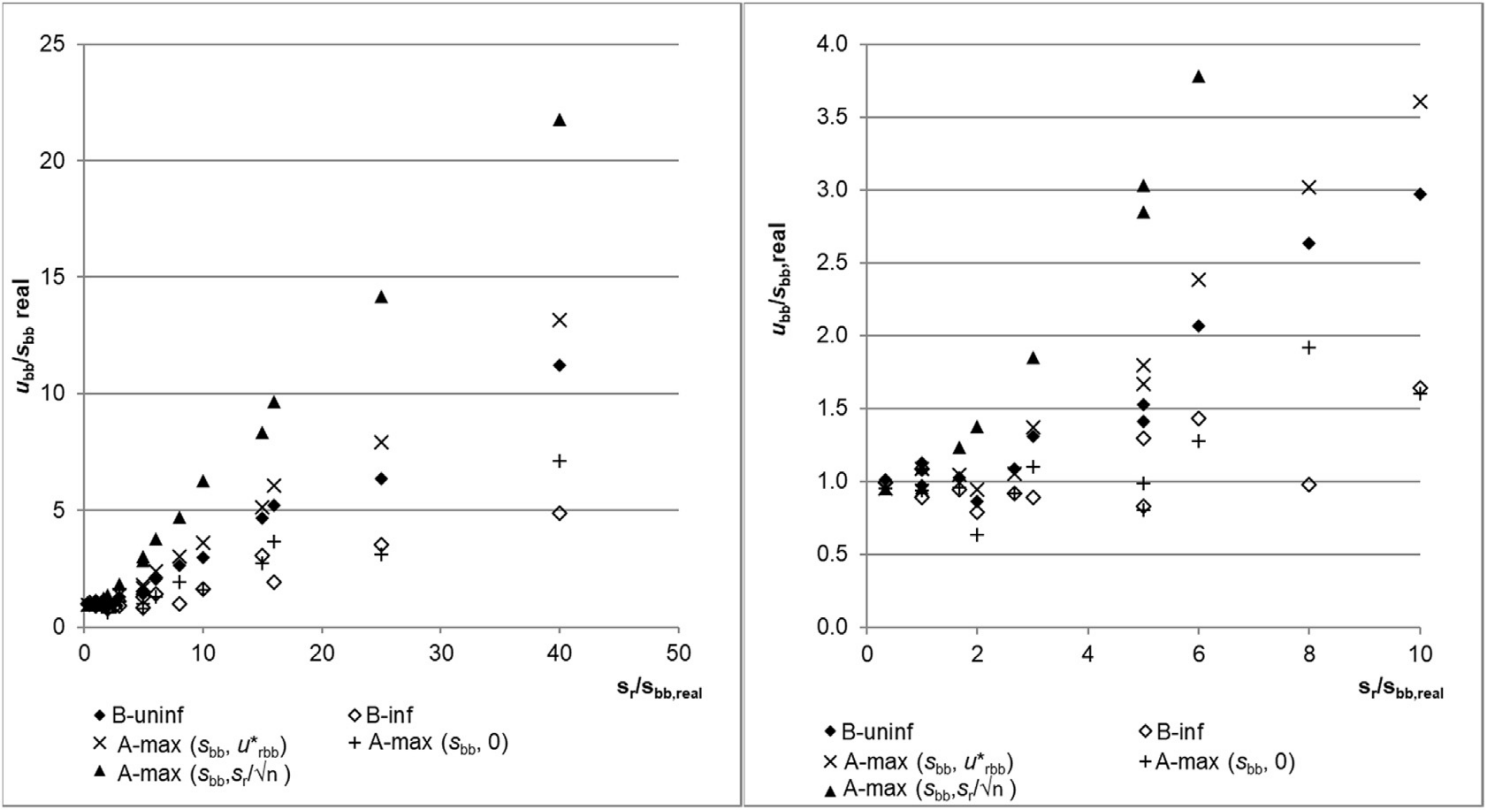
Average estimation. Left all s_r_/s_bb,real_ ratios. Right: only area for s_r_/s_bb,real_ up to 10.

The plot of the average estimates of the five evaluation approaches is shown in Fig. 3. The graphs show that using A-max(*s*_bb_, *s*_r_/√n) is for high *s*_r_/*s*_bb,real_ ratios, which is significantly biased towards high results. This is expected and known, especially for higher *s*_r_/*s*_bb,real_ ratios where the standard deviation of the means only reflects the random variation of the averages due to repeatability. Also, as expected, A-max(*s*_bb_,0) shows a very low average bias. This is because for a perfectly homogeneous material (*s*_bb,real_ = 0) in half of the cases, *MS*_between_ < *MS*_within_ and the value 0 is in these cases the correct estimate. Using Bayesian statistics with an informative prior (B-inf) performs on average, similarly well as A-max(s_bb_,0). The on the average high positive bias of Bayesian analysis with a diffuse prior is somewhat surprising. On average, the positive bias is comparable to A-max(*s*_bb,_*u*∗_bb_), which only reflects measurement repeatability and the study setup. As is evident from the equations, the average positive bias for A-max(*s*_bb,_*u*∗_bb_) is smaller than for A-max(*s*_bb_, *s*_r_/√n) due to the inclusion of the term reflecting the degrees of freedom of *s*_r_.

The finding that the results from Bayesian analysis (both using diffuse and informative priors) are lower than when using the maximum of *s*_bb_ and *u*∗_bb_ seemingly contradicts [10]. This publication stated, for the examples investigated that “Bayesian analysis gives a value for the between-unit standard deviation, which is not even close to *u*∗_bb_”. Several factors explain this apparent contradiction: On the one hand, the ratios of *s*_r_/*s*_bb_ explored in Ref. [10] range from 0.4 to 3.4 which is significantly lower than the ranges explored here and in the majority (7 of 9) cases, *s*_bb_ was lower than *s*_r_. In addition, 6 replicates per unit were performed. This means, as shown by the fact that *s*_bb_ could be evaluated in all cases by ANOVA, that there was not a single case where *u*∗_bb_ was larger than *s*_bb_. A comparison of the estimate for *s*_bb_ and *u*∗_bb_ is not relevant, as *u*∗_bb_ should not be used as an estimate of *u*_bb_ in such cases.

#### 3.2.2 Maximum overestimation

**Fig. 4:**
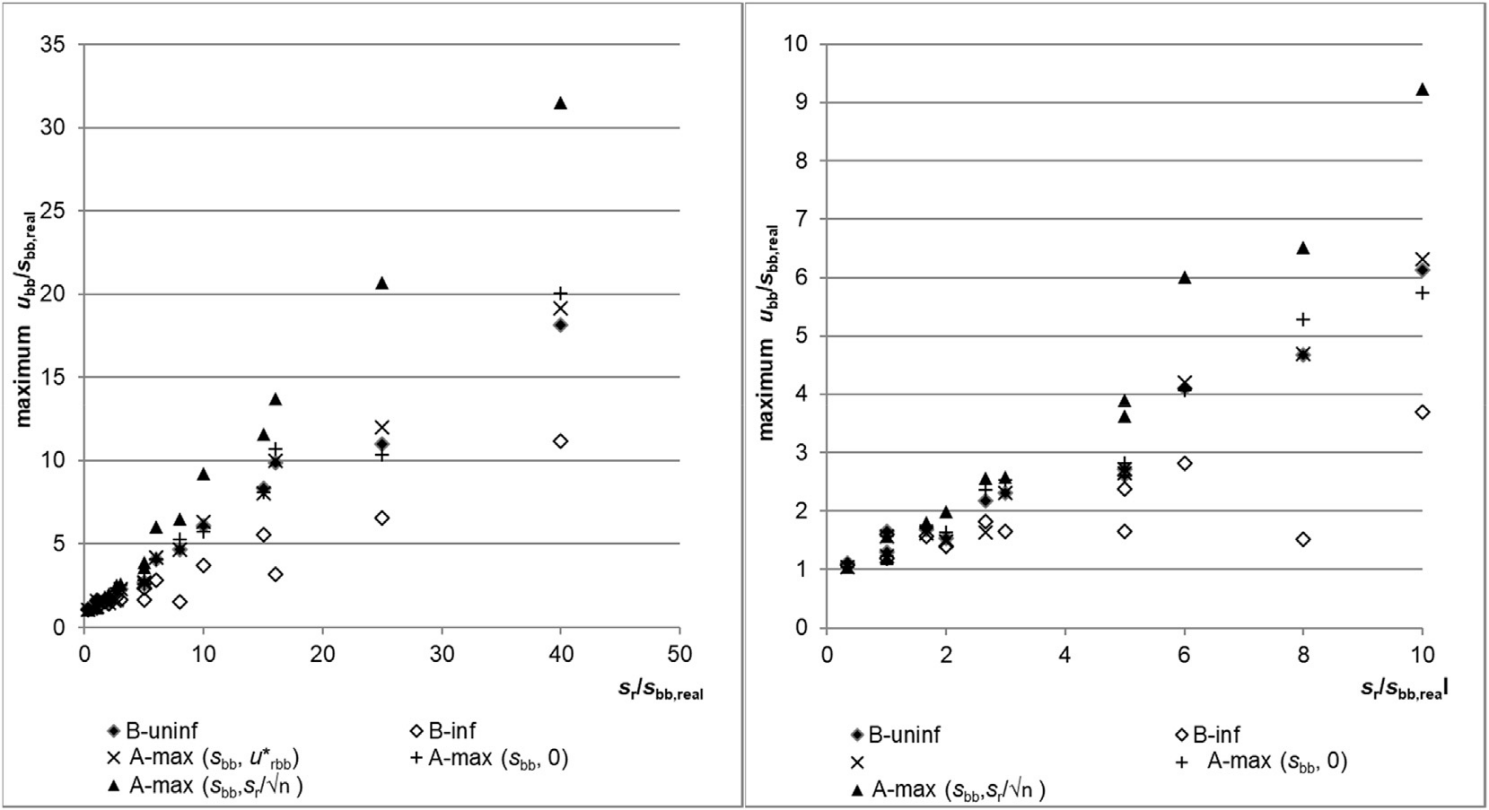
Maximum overestimation of s_bb,real_. Left all s_r_/s_bb,real_ ratios. Right: only area for s_r_/s_bb,real_ up to 10.

In general, both CRM producers and users are less interested in the average bias than in the bias introduced for a specific CRM. The evaluation of the maximum overestimation by the five approaches is shown in Fig. 4. The general trend for the maximum overestimation is the same as for the average estimate discussed before: A-max(*s*_bb_, *s*_r_/√n) results in the highest and B-inf in the lowest maximum overestimation. However, it is noticeable that the differences between the various approaches is less pronounced than in the average estimation. Especially for *s*_r_/s_bb_ ratios below 6, the results from the various approaches are rather close together. This can be explained by the fact that *s*_r_ also generates a random variation between units. Whenever this variation coincides with the real between-unit variation (i.e. the average of units with a higher average increases even more by random fluctuations), a too high estimate for *s*_bb_ will be given and cannot be reduced by any evaluation approach. The severity can only be mitigated by performing more measurements per unit or by measuring more units: more measurements per unit makes the measured unit mean a better estimate of the real unit mean while measuring more units reduces the probability that this enhancement occurs for all units.

#### 3.2.3 Maximum underestimation

**Fig. 5:**
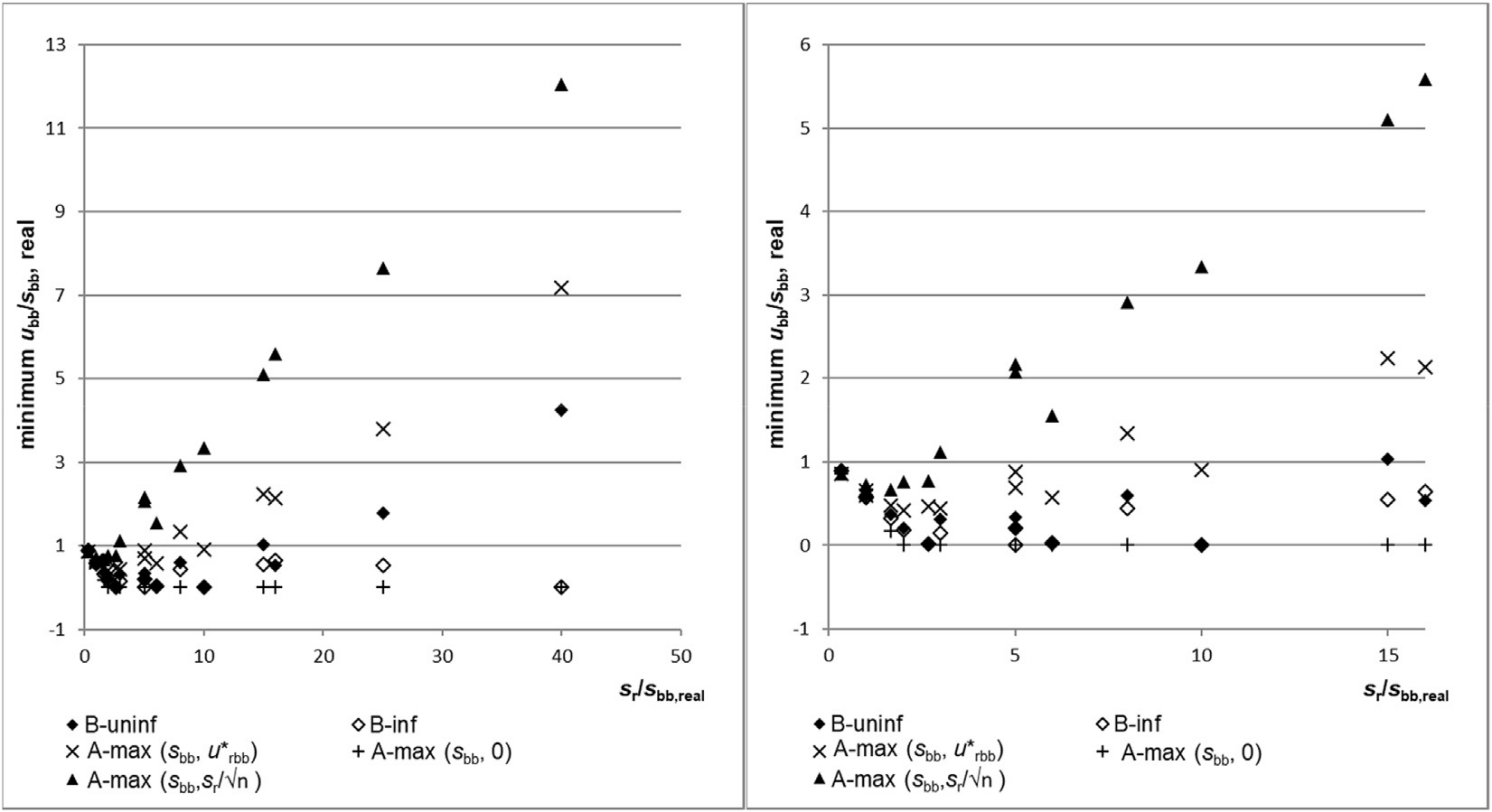
Maximum underestimation of s_bb,real_. Left all s_r_s_bb, real_ ratios. Right: only area for s_r_/s_bb,real_ up to 16. Numbers below 1 correspond to an underestimation of the real between-unit variation; values above 1 show that even the lowest estimate of u_bb_ was above s_bb,real_.

The maximum underestimation observed for the homogeneity studies investigated is shown in Fig. 5. In this graph, values below 1 show that a particular approach is expected to underestimate *s*_bb,real_ for a certain *s*_r/_s_bb,real_ combination in 5% of the cases. Values above 1 show that for a given *s*_r_/_sbb,real_ combination *u*_bb_ should in 95% of the cases overestimate *s*_bb, real_. The graph shows that using A-max(*s*_bb_, *s*_r_/√n) is of all the investigated approaches, the least prone to severe underestimations of s_bb,real_. The minimum ratio *u*_bb_/*s*_bb,real_ was 0.61, i.e. an underestimation by only 39%. This safe estimate, of course, comes at a price: Not only is the average result biased (see above), but also from *s*_r_/s_bb,real_ ratios of 5 results closest to the real value still overestimate *s*_bb,real_ by a factor of about two or more.

The other extreme is as expected, A-max(s_bb_,0): Only for *s*_r_/s_bb,real_ ratios of 1 or below was *MS*_between_ for all 20 simulated studies, which are larger than *MS*_within_, leading to small to modest underestimations. However, at higher ratios for *s*_r_/s_bb,real_, *MS*_between_ was at least in one of the 20 simulated studies smaller than *MS*_within_, which resuled in an estimate of *u*_bb_ of 0 and hence a complete underestimation of s_bb,real_. This results in lower confidence limits of 0 for all *s*_bb_/*s*_r_ ratios above 1.7.

The lower confidence limit for A-max(*s*_bb_,*u*∗_bb_) is between 0.4 and 0.6 ratios of *s*_r_/_sbb,real_ below 5, indicating that underestimations by 40–60% are possible. The notable exception is the combination, *s*_bb_ = 3 and *s*_r_ = 8, where the lower confidence limit is only 0.07, corresponding to an underestimation of 93%. However, it should be pointed out that a single overestimation of *s*_bb_ by a factor of 3 causes this lower confidence limit, which increased the standard deviation and hence resulted in broad confidence bands. The lowest value observed was 0.64 (corresponding to an underestimation of 36%). In most of the *s*_r_/*s*_bb,real_ combinations, the calculated *s*_bb_ was still larger than *u*∗_bb_. This means the underestimation was caused by the compensation of the between-unit variation involving the random fluctuations due to measurement repeatability. In all of these cases, the underestimation was still slightly less severe than when applying Bayesian statistics (see below). The findings that *u*∗_bb_ sometimes underestimates *s*_bb,real_ partly supports the criticism of the approach raised in Ref. [7] that *u*∗_bb_ may underestimate the potentially hidden homogeneity. While these simulated data confirm this worry, they also indicate that this is only a problem for low *s*_r_/s_bb,real_ combinations, i.e. for either very inhomogeneous materials or very repeatable methods. The maximum underestimation observed in these cases was 50%, which can have a substantial impact on the assigned uncertainty (see section “Potential impact of the under/overestimation of *u*_bb_). On the other hand, for all *s*_r_/*s*_bb,real_ ratios larger or equal than 8, even the lower confidence limit of A-max(*s*_bb_,*u*∗_bb_) is an overestimation of the s_bb,real_, which is driven by *u*∗_bb_. This indicates that for higher ratios of *s*_r_/*s*_bb,real_ u∗_bb_ is a conservative estimate of *s*_bb,real_.

As the between-unit variation is in many of the investigated scenarios hidden by the random variation, the performance of Bayesian analysis with respect to the maximum underestimation is mixed. At *s*_r_/*s*_bb,real_ ratios above 20, using the diffuse prior results in all cases in moderate overestimations, whereas the use of the informative prior does not lead to an overestimation. On the other hand, using a point estimate for *s*_bb_ from Bayesian analysis cannot prevent significant underestimation of *u*_bb_ either. In all cases, the maximum underestimations of *u*_bb_ of the Bayesian approaches were worse than A-max(*s*_bb_,*u*∗_bb_): *u*_bb_ was underestimated by up to about 65% by both informative and diffuse priors. The reason here is the opposite of the one discussed under “Maximum overestimation”: Whenever the random fluctuation of unit means, caused by method repeatability, compensates for the real variation of unit means, *u*_bb_ will be underestimated. As in the case of maximum overestimation, also the occurrence of underestimation can only be reduced by either performing more measurements per unit or performing measurements on more units.

As noted above, in cases where *s*_r_/*s*_bb_ >1, the posterior distributions from Bayesian analysis are very broad, and it could be argued that using the median is a poor point estimate for *s*_bb_. However, investigation of other point estimates is beyond the scope of this manuscript.

#### 3.2.4 Fraction of significant underestimations

**Fig. 6:**
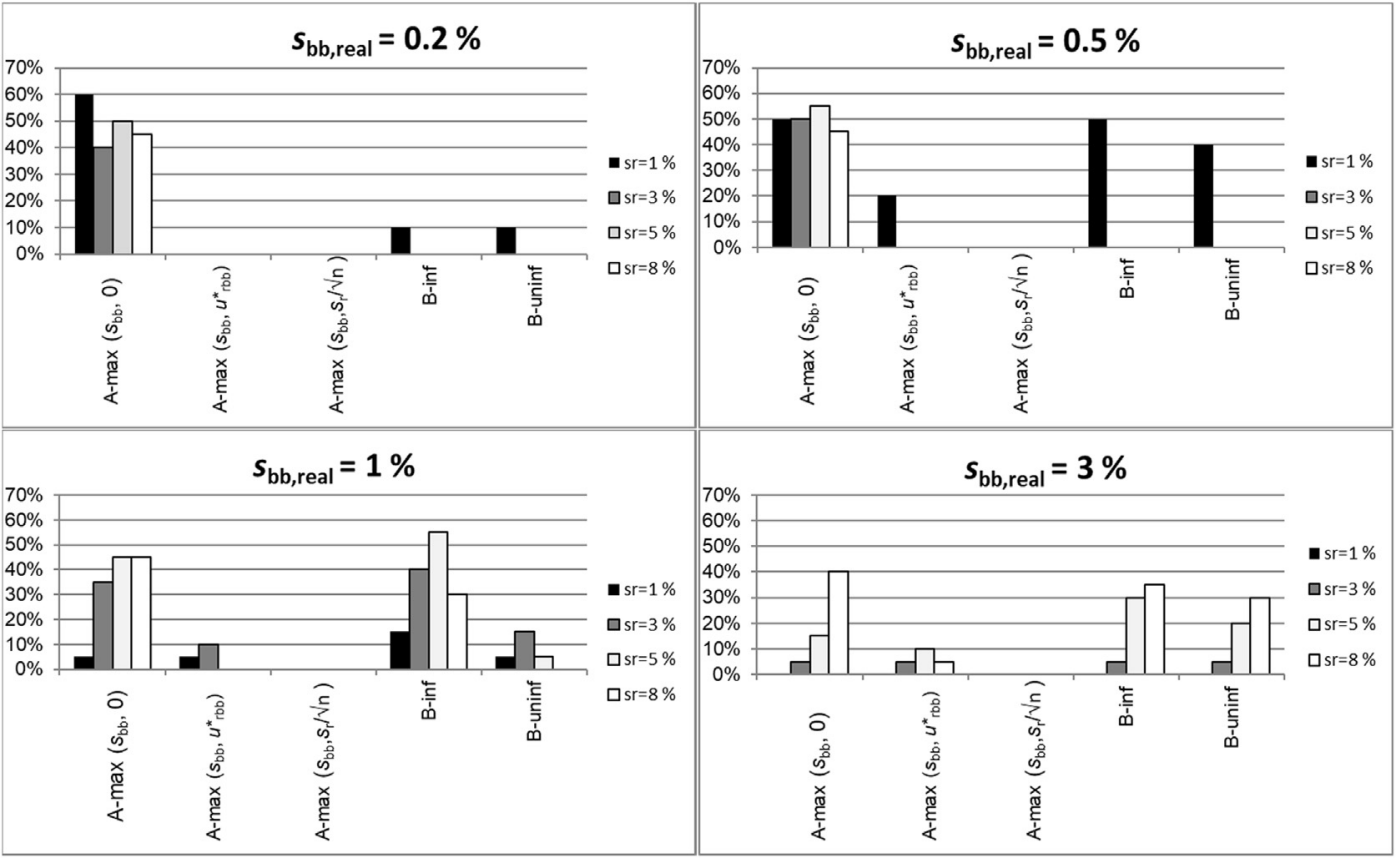
Fraction of cases where u_bb_ was underestimated by more than 30%.

The fractions of cases where *u*_bb_ was underestimated by more than 30% are shown in Fig. 6. As expected from the discussion above, the extremes are again formed by A-max(*s*_bb_,0) and A-max(*s*_bb_, *s*_r_/√n): Using A-max(*s*_bb_, *s*_r_/√n) resulted in none of the 320 simulated homogeneity studies in an underestimation of *u*_bb_ by more than 30%. This confirms the suggestion made in Ref. [8] that the *s*_means_ is a safe estimate of *u*_bb_. On the other hand, using max(*s*_bb_,0) as an estimate for *u*_bb_ resulted in half of the cases for *s*_bb,real_ of 0.2 and 0.5 of underestimations of at least 30%. The outcome was slightly better for *s*_bb,real_ = 1, but even there, 35–45% of *u*_bb_ were underestimated by more than 30% when *s*_r_ was ≥3%. This shows that using max(*s*_bb_,0) as an estimate of *u*_bb_ results, in many cases, in significant underestimations of the uncertainty unless the requirement for method repeatability of ISO Guide 35 is met. It should be noted that the respective section of ISO Guide 35 (section 7.7.3) refers to this requirement, albeit only in a note. Seeing the high probability of underestimating the assigned uncertainty, making this requirement more prominent would seem a reasonable suggestion for a future revision of this guide.

A-max(*s*_bb_,*u*∗_bb_) resulted in 11 of the 320 simulated studies in underestimations of *u*_bb_ by at least 30%. This low rate is because the studies investigated have rather high ratios of *s*_r_/s_bb,real_ where at high *s*_r_, *u*∗_bb_ exceeds the assumed *s*_bb,real_. The situation would have been less positive for lower ratios of *s*_r_/*s*_bbreal_ where *s*_bb_ can usually be calculated, but *u*∗_bb_ becomes too small to account for the potentially hidden inhomogeneity. This rather low fraction of severe underestimations relativizes the concern of *u*∗_bb_ not being a sufficiently conservative estimate of the potentially hidden inhomogeneity raised in Ref. [7]: while the data confirm that *u*∗_bb_ may underestimate the true hidden inhomogeneity, they also indicates that the underestimation is limited and rather rare for the conditions studied.

Using Bayesian analysis does not mitigate the risk of severely underestimating *u*_bb_. There was hardly any difference in the percentage of significant underestimation between the use of informative and diffuse priors. For some combinations of *s*_r_/*s*_bb,real_, *u*_bb_ was underestimated by more than 30% in 40% and more of the studies. For all combinations of *s*_r_/*s*_bb,real_ investigated, Bayesian analysis performed worse than A-max(*s*_bb_,*u*∗_bb_) using this measure, with underestimations in 26 (diffuse prior) and 54 (informative prior) of the 320 simulated studies.

#### 3.2.5 Potential impact of the under/overestimation of u_bb_

The discussion above focussed on over- and underestimation of *u*_bb_. However, *u*_bb_ in isolation is only relevant for non-certified reference materials where the material information sheet must indicate the degree of homogeneity. For certified reference materials, *u*_bb_ is always combined with the *u*_char_ and the uncertainties of long-term and short-term stability (*u*_lts_, *u*_sts_ estimated either from previous knowledge or from dedicated stability studies, as described for example in Ref. [15]) to obtain *u*_CRM_, which is calculated as shown in Equation (8).

Equation 8 $$u_{CRM}=\sqrt{u_{bb}^{2}+u_{char}^{2}+u_{lts}^{2}+u_{sts}^{2}}.$$

**Fig. 7:**
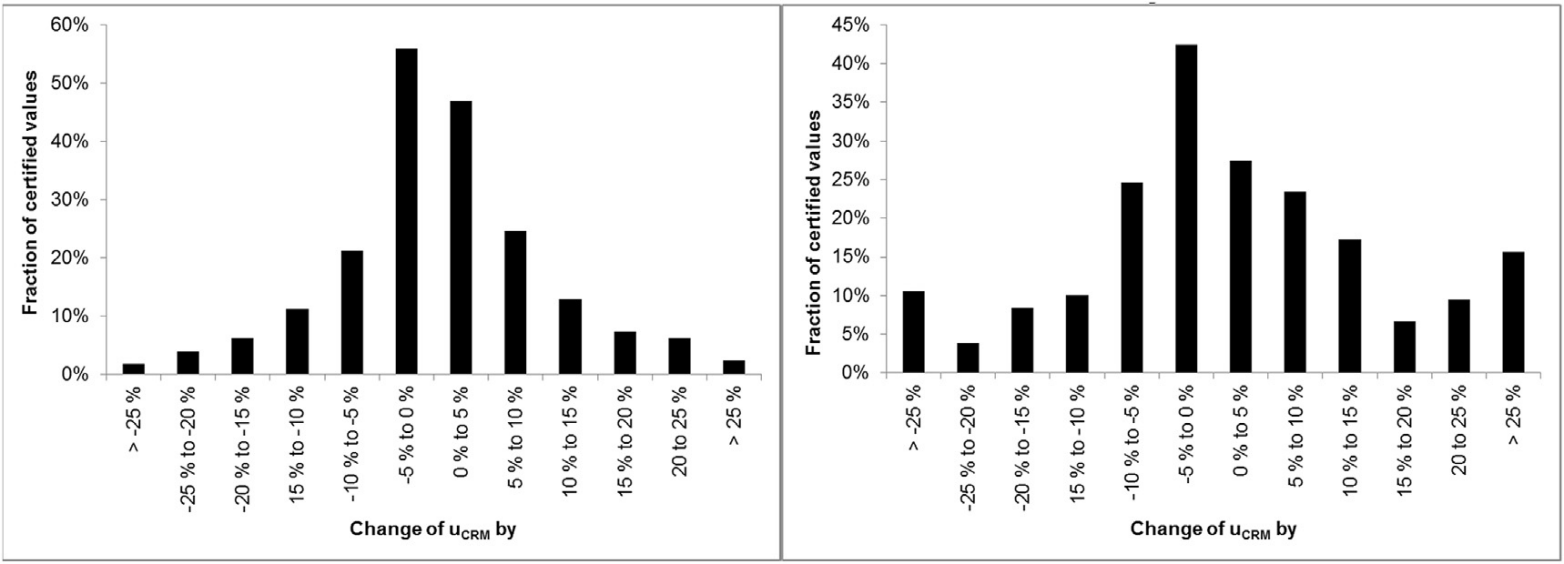
Effect on u_CRM_ of a change of u_bb_ by 30% (left) and 50% (right). Only u_bb_ and u_char_ were considered for the estimation of u_CRM_ for this analysis.

Depending on the sizes of *u*_char_,*u*_bb_, *u*_lts_ and *u*_sts_ relative to each other, an over- and underestimation of *u*_bb_ may have a significant effect or no effect at all on *u*_CRM_. In investigating the effect of a false estimation of *u*_bb_ on certified uncertainties in a real-life situation, the effect of an increase/decrease of the *u*_bb_ used for the certified values investigated in the section “ Need for an uncertainty estimation” by 30% and 50% was investigated. In this case, *u*_lts_, *u*_sts_ were not included in the calculation as different CRM producers use widely different approaches to account for these uncertainties. The results, depicted in Fig. 7, seems at first glance to indicate that the effects of overestimation of *u*_bb_ are more pronounced than of underestimation. This is because in most cases, *u*_bb_ is smaller than *u*_char_, so a further reduction has hardly any effect. For the certified values investigated here, even changes of *u*_bb_ by ± 50% have a rather small effect. For more than 75% of the values, the change of *u*_CRM_ is below 10%. In reality, the effect is even smaller as the JRC has, in line with the requirements of ISO 17034, the policy to include quantitative estimates of *u*_lts_ and *u*_sts_ into *u*_CRM_. This seemingly higher importance of overestimation, however, needs to be contrasted with the rounding of certified uncertainties. The policy for CRMs produced at the JRC is never to round down uncertainties (e.g. 0.112 is rounded up to 0.12) and to round uncertainties such that the rounding error is within 3 and 30% of the uncertainty. This means that for the cases studied, an overestimation of *u*_bb_ by 30% would virtually never have resulted in an overestimation of *u*_CRM_ by more than the accepted rounding error. Also, an overestimation of *u*_bb_ by 40% would only in a few cases have led to such an overestimation. On the other hand, underestimation of *u*_bb,_ even by 30%, would have in 12% of the cases resulted in underestimations by more than 15%.

Fig. 7 shows the aggregate result over all analytes investigated, but the result is the same when limiting the investigation to certified element mass fractions (data not shown).

## 4 Conclusions

Given the results of the evaluation of real and simulate homogeneity studies, the following conclusions are taken:

Even using state of the art methods, the actual homogeneity of matrix reference materials, combined with the limitations on the amount of material per unit and the resources that can be put into a single homogeneity study, estimation of *u*_bb_ by one-way ANOVA will in many cases not be possible. Also, the limit for method repeatability as given in ISO Guide 35:2017 is, in many cases, impossible to meet. There is, therefore, a real need to find reliable approaches to deal with cases where *MS*_between_ < *MS*_within_. While all the approaches investigated in this work have their specific strengths and shortcomings, it must be borne in mind that this study deliberately investigated cases where the analytical noise overpowers the signal from the between unit variation, hence examining cases where there was not enough “data” in the numbers to perform good statistics.

Setting *u*_bb_ zero when *MS*_between_ < *MS*_within_ results in many cases in severe underestimations of *u*_bb_. It seems appropriate that future revisions of ISO Guide 35 should strongly emphasise the fact that this estimate is only valid if the repeatability limit is fulfilled, otherwise the maximum of (*s*_bb_,0) is not a suitable estimate for the between-unit variation.

Estimating *u*_bb_ as A-max(*s*_bb_, *s*_r_/√n) was, as found in all cases investigated here, to guard against severe underestimations of *u*_bb_. This, however, comes at the cost of severe overestimations at already rather moderate ratios of *s*_bb_/*s*_r_, which diminishes the value of the CRMs for the users.

Estimating *u*_bb_ as the maximum of (*s*_bb_, *u*∗_bb_) was found in some cases, especially at low s_bb_/*s*_r_ ratios to underestimate *s*_bb_, hence confirming the suspicion raised in Ref. [7]. On the other hand, it mitigated the occurrence and extent of severe underestimations. Also, this advantage comes at the price at on average positive biases towards too high values, albeit lower ones than seen for A-max(*s*_bb_, *s*_r_/√n).

Using the median as point estimates from Bayesian analysis is not a universal solution to the problem of estimating *s*_bb_ when *s*_r_ » *s*_bb_, because neither using informative nor uninformative priors prevented severe underestimations of *u*_bb_. It might be that other point estimates could distribution mitigates the risk of underestimations. This is an interesting option, which should be investigated further. The use of informative priors, however, limit the positive bias of the estimation.

A solution to cases where *s*_r_ » *s*_bb_ could be implementing a risk-based approach as advocated in ISO 17034: After estimating *u*_bb_ (by whatever approach), *u*_lts_, *u*_sts_ and *u*_char_ it can be determined what the effect of a severe over- or underestimation of *u*_bb_ on *u*_CRM_ could have. If *u*_bb_ is a minor component, a large relative bias of *u*_bb_ will not have a significant effect on *u*_CRM_. If *u*_bb_ is significant, the reference materials producer can (preferably, considering past experience) decide which risk of over/underestimation the actual uncertainty is acceptable and choose an evaluation approach that limits this effect.

## CRediT authorship contribution statement

**Thomas P.J. Linsinger:** Conceptualization, Data curation, Formal analysis, Investigation, Methodology, Visualization, Writing - original draft, Writing - review & editing.

## Declaration of competing interest

The authors declare that they have no known competing financial interests or personal relationships that could have appeared to influence the work reported in this paper.
