# Supplementary material for: Evaluation of CRM homogeneity in cases of insufficient method repeatability: Comparison of Bayesian analysis with substitutes for ANOVA based estimates
Source: Anal Chim Acta X. 2020 Apr 14;5:100049. doi: 10.1016/j.acax.2020.100049 (PMC7587033; doi:10.1016/j.acax.2020.100049)
Supplement: Multimedia component 1 [file mmc1.docx]

**Supplementary material to Evaluation of CRM homogeneity in cases of insufficient method repeatability: comparison of Bayesian analysis, ANOVA and the average of unit means**

The table below lists the CRMs used in the section " Need for an uncertainty estimation for alternative homogeneity evaluations and practicality of a repeatability limit", their bibliographical reference and the source, where the certification report is available

| **CRM code** | **Description** | **Citation of the certification report** | **Weblink of the certification report** |
| --- | --- | --- | --- |
| ERM-AD442k | concentration of lamda-DNA in buffer | Pui Yan Jenny Chung *et al.* , The certification of the mass of lambda DNA in a solution Certified Reference Material: ERM®-AD442k, EUR 27160 EN, Luxembourg: Publications Office of the European Union, 2015 | <https://crm.jrc.ec.europa.eu/p/ERM-AD442k> |
| ERM-AD453k/IFCC | enzymatic activity of lactate dehydrogenase | J.Kuhlmann, B. Toussaint, H. Schimmel, H. Emons, The certification of the catalytic activity concentration of lactate dehydrogenase in ERM®-AD453k/IFCC, EUR 28098 EN, Luxembourg: Publications Office of the European Union, 2016, ISBN 978-92-79-61782-9 | <https://crm.jrc.ec.europa.eu/p/ERM-AD453k_IFCC> |
| ERM-AD454k/IFCC | enzymatic activity of alanine aminotransferase | J. Kuhlmann, B. Toussaint, H. Schimmel, H. Emons, The certification of the catalytic activity concentration alanine aminotransferase in ERM®-AD454k/IFCC, EUR 28105 EN, Luxembourg: Publications Office of the European Union, 2016, ISBN 978-92-79-61791-1 | <https://crm.jrc.ec.europa.eu/p/ERM-AD454k_IFCC> |
| ERM-AD455k/IFCC | enzymatic activity of creatine kinase | J. Kuhlmann, B. Toussaint, H. Schimmel, H. Emons, The certification of the catalytic activity concentration of creatine kinase in ERM®-AD455k/IFCC, EUR, EUR 28101 EN, Luxembourg: Publications Office of the European Union, 2016, ISBN 978-92-79-61786-7 | <https://crm.jrc.ec.europa.eu/p/ERM-AD455k_IFCC> |
| ERM-AD483a | copy number concentration of a plasmid in buffer | J. Mátrai *et al.*, A set of three plasmid DNA calibration solutions bearing a porcine-specific DNA fragment Certified Reference Materials: ERM®-AD483a, ERM®-AD483b, ERM®-AD483c, EUR 27505 EN, Luxembourg: Publications Office of the European Union, 2015, ISBN 978-92-79-52306-9 | <https://crm.jrc.ec.europa.eu/p/ERM-AD483> |
| ERM-AD483b | copy number concentration of a plasmid in buffer |  |  |
| ERM-AD483c | copy number concentration of a plasmid in buffer |  |  |
| ERM-AD500/IFCC | glycaeted haemoglobin in buffer | A. Muñoz *et al.*, Certification of the amount-of-substance fraction of HbA1c versus the sum of HbA0 and HbA1c inhaemoglobin: ERM®- AD500/IFCC, EUR 27574 EN, Luxembourg: Publications Office of the European Union, 2016, ISBN 978-92-79-53878-0 | <https://crm.jrc.ec.europa.eu/p/ERM-AD500_IFCC> |
| ERM-BB185 | trace elements in bovine liver | H. Leys, H. Emteborg, J. Seghers, J. Snell, The certification of the mass fractions of elements in bovine liver: ERM®-BB185, EUR 29623 EN, Luxembourg: Publications Office of the European Union, 2019, ISBN 978-92-79-98881-3 | <https://crm.jrc.ec.europa.eu/p/ERM-BB185> |
| ERM-BC717 | nivalenol and deoxynivalenol in maize | A. Veršilovskis, A. Bernreuther, The additional certification of the mass fractions of deoxynivalenol and nivalenol in maize: ERM®-BC717, EUR 26502 EN, Luxembourg: Publications Office of the European Union, 2014, ISBN 978-92-79-35454-0 | <https://crm.jrc.ec.europa.eu/p/ERM-BC717> |
| ERM-BD512 | trace elements in chocolate | J. Snell *et al.*, The certification of the mass fractions of cadmium copper, manganese and nickel in dark chocolate: ERM®- BD512, EUR 28936 EN, Luxembourg: Publications Office of the European Union, 2018, ISBN 978-92-79-77066-1 | <https://crm.jrc.ec.europa.eu/p/ERM-BD512> |
| ERM-CA100 | polycyclic aromatic hydrocarbons in water | I. Dosis *et al.*, The certification of the concentration of Polycyclic Aromatic Hydrocarbons (PAHs) in surface water: ERM®-CA100, EUR 27964 EN, Luxembourg: Publications Office of the European Union, 2015, ISBN 978-92-79-59110-5 | <https://crm.jrc.ec.europa.eu/p/ERM-CA100> |
| ERM-CA403 | trace elements in seawater | R. Sánchez *et al.*, The certification of the mass concentration of As, Cd, Co, Cu, Mn, Mo, Ni and Pb, and density in seawater: ERM®- CA403, EUR 29055 EN, Luxembourg: Publications Office of the European Union, 2018, ISBN 978-92-79-77706-6 | <https://crm.jrc.ec.europa.eu/p/ERM-CA403> |
| ERM-CC144 | total element content in sewage sludge | A. Santoro, T. Venelinov, A. Oostra, A. Held, The Certification of the Mass Fraction of the Total Content and the Aqua Regia Extractable Content of As, Cd, Co, Cr, Cu, Fe, Hg, Mn, Ni, Pb and Zn in Sewage Sludge Certified Reference Material ERM®-CC144, EUR 25026 EN, Luxembourg: Publications Office of the European Union, 2011, ISBN 978-92-79-21878-1 | <https://crm.jrc.ec.europa.eu/p/ERM-CC144> |
| ERM-CC537a | brominated flame retardants in sediment | M. Ricci, H. Emteborg, J. Seghers, A. Held, The certification of the mass fractions of polybrominated diphenyl ethers (PBDEs) and α-, β- and γ-hexabromocyclododecane (HBCD) in freshwater sediment: ERM®-CC537a, EUR 28880 EN, Luxembourg: Publications Office of the European Union, 2018, ISBN 978-92-79-76482-0 | <https://crm.jrc.ec.europa.eu/p/ERM-CC537a> |
| ERM-CD200 | trace elements in seaweed | A. Santoro *et al.*, The certification of the mass fraction of the total content of As, Cd, Cu, Hg, Pb, Se and Zn in Bladderwrack (Fucus vesiculosus): ERM- CD200, EUR 26525 EN, Luxembourg: Publications Office of the European Union, 2014, ISBN 978-92-79-35531-8 | <https://crm.jrc.ec.europa.eu/p/ERM-CD200> |
| ERM-CE100 | hexachlorobenzene and hexachlorobutadiene in fish | I. Dosis *et al.*, The certification of the mass fractions of hexachlorobenzene (HCB) and hexachlorobutadiene (HCBD) in fish tissue: ERM®-CE100, EUR 27965 EN, Luxembourg: Publications Office of the European Union, 2016, ISBN 978-92-79-59111-2 | <https://crm.jrc.ec.europa.eu/p/ERM-CE100> |
| ERM-DA470k/IFCC | beta-2 microglobulin in human serum | G. Auclair *et al.*, The certification of the mass concentration of beta-2-microglobulin in human serum:ERM-DA470k/IFCC, EUR 26972 EN, Luxembourg: Publications Office of the European Union, 2015, ISBN 978-92-79-44555-2( | <https://crm.jrc.ec.europa.eu/p/ERM-DA470k_IFCC> |
| ERM-DA476/IFCC | anti-MPO IgI in human serum | E. Monogioudi *et al.*, The certification of anti-myeloperoxidase immunoglobulin G in human serum ERM® - DA476/IFCC, EUR 27092 EN, Luxembourg: Publications Office of the European Union, 2015, ISBN 978-92-79-45349-6( | <https://crm.jrc.ec.europa.eu/p/ERM-DA476_IFCC> |
| ERM-DA482a/IFCC | abeta 42 in human cerebrospinal fluid | J. Kuhlmann *et al.*, The certification of Amyloid β1-42 in CSF in ERM®-DA480/IFCC, ERM®-DA481/IFCC and ERM®-DA482/IFCC, EUR 28691 EN, Luxembourg: Publications Office of the European Union, 2017, ISBN 978-92-79-70556-4 ( | <https://crm.jrc.ec.europa.eu/p/ERM-DA482_IFCC> |
| ERM-DA482b/IFCC | abeta 42 in human cerebrospinal fluid |  |  |
| ERM-DA482c/IFCC | abeta 42 in human cerebrospinal fluid |  |  |
| ERM-DA483/IFCC | anti-PR3 IgG in human serum | E. Monogioudi *et al.*, The certification of the mass concentration of immunoglobulin G proteinase 3 anti-neutrophil cytoplasmic autoantibodies (IgG PR3 ANCA) in human serum: ERM® - DA483/IFCC, EUR 28537 EN, Luxembourg: Publications Office of the European Union, 2017, ISBN 978-92-79-66974-3 | <https://crm.jrc.ec.europa.eu/p/ERM-DA483_IFCC> |
| ERM-EB074B | trace elements in copper | T. Bacquart, T. P.J. Linsinger, The certification of trace elements mass fraction in  electrolytic copper: ERM®-EB074A, B and C, EUR 26890 EN, Luxembourg: Publications Office of the European Union, 2015, ISBN 978-92-79-43606-2 | <https://crm.jrc.ec.europa.eu/p/ERM-EB074B> |
| ERM-EB075B | trace elements in copper | T. Bacquart, T. P.J. Linsinger, The certification of trace elements mass fraction in  electrolytic copper: ERM®-EB075A, B and C, EUR 26870 EN, Luxembourg: Publications Office of the European Union, 2015, ISBN 978-92-79-43216-3 | <https://crm.jrc.ec.europa.eu/p/ERM-EB075B> |
| ERM-EB530 | gold in aluminium | T. Bacquart, A. Moens, T. Linsinger, The certification of the gold mass fraction in Al-0.1%Au alloy: ERM®-EB530A, B and C, EUR 26830 EN, Luxembourg: Publications Office of the European Union 2014, ISBN 978-92-79-40180-0 | <https://crm.jrc.ec.europa.eu/p/ERM-EB530> |
| ERM-EC680m | trace elements in plastics | R. Sanchez Romero, T. P.J. Linsinger, The Certification of the Mass Fractions of As, Br, Cd, Cr, Hg, Pb, S, Sb, Sn and Zn in Low-Density Polyethylene: ERM®-EC680m, EUR 27572 EN, Luxembourg: Publications Office of the European Union, 2015, ISBN 978-92-79-53800-1 | <https://crm.jrc.ec.europa.eu/p/ERM-EC680m> |
| ERM-EC681m | trace elements in plastics | R. Sanchez Romero, T. P.J. Linsinger, The Certification of the Mass Fractions of As, Br, Cd, Cr, Hg, Pb, S, Sb, Sn and Zn in Low-Density Polyethylene: ERM®-EC681m, EUR 27576 EN, Luxembourg: Publications Office of the European Union, 2015, ISBN 978-92-79-53881-0 | <https://crm.jrc.ec.europa.eu/p/ERM-EC681m> |
| ERM-EF001 | various parameters of biodiesel | M. Ulberth-Buchgraber *et al.*, The certification of the mass fraction of the ester, linolenic acid methyl ester, monoglyceride, diglyceride, triglyceride, total glycerol and water content, density, viscosity, oxidation stability, acid value, iodine value and flash point of biodiesel: ERM®- EF001, EUR 26711 EN, Luxembourg: Publications Office of the European Union, 2014. ISBN 978-92-79-38971-9 | <https://crm.jrc.ec.europa.eu/p/ERM-EF001> |
| ERM-EF003 | various parameters in a diesel-biodiesel mixture | M. Ulberth-Buchgraber, J. Charoud-Got, H. Emteborg, A. Held, The certification of selected chemical and physical properties in automotive diesel fuel containing a volume fraction of 7 % biodiesel: ERM®- EF003, EUR 28864 EN, Luxembourg: Publications Office of the European Union, 2018, ISBN 978-92-79-74143-2 | <https://crm.jrc.ec.europa.eu/p/ERM-EF003> |
| ERM-EF318k | solvent yellow 124 in diesel | S. Elordui-Zapatarietxe Del Aguila, J.Charoud-Got, A.Held, H. Emteborg, The certification of mass fraction of Solvent Yellow 124 in gas oil: ERM®- EF318k, EUR 29266 EN, Luxembourg: Publications Office of the European Union, 2018, ISBN 978-92-79-87864-0 ( | <https://crm.jrc.ec.europa.eu/p/ERM-EF318k> |
| ERM-FD069 | size distribution of corundum | T.Linsinger, J. Charoud-Got, V. Kestens, T.Gerganova, The certification of particle size distribution of corundum: ERM®- FD069, EUR 28955 EN, Luxembourg: Publications Office of the European Union, 2018, ISBN 978-92-79-77158-3 | <https://crm.jrc.ec.europa.eu/p/ERM-FD069> |
| ERM-FD101b | size of nanoparticles | Y. Ramaye *et al.*, The certification of equivalent diameters of silica nanoparticles in aqueous solution: ERM®-FD101b, EUR 28362 EN, Luxembourg: Publications Office of the European Union, 2017, ISBN 978-92-79-64637-9 | <https://crm.jrc.ec.europa.eu/p/ERM-FD101b> |
| ERM-FD102 | size of nanoparticles | V. Kestens, G. Roebben, The certification of equivalent diameters of a mixture of silica nanoparticles in aqueous solution: ERM- FD102, EUR 26656 EN, Luxembourg: Publications Office of the European Union, 2014, ISBN 978-92-79-38396-0 | <https://crm.jrc.ec.europa.eu/p/ERM-FD102> |
| IRMM-427 | perfluoroalkyl substances in fish | M. Dabrio *et al.*, Certification of the mass fraction of perfluoroalkyl substances (PFASs) in fish tissue (pike-perch): IRMM-427 , EUR 27429, Luxembourg: Publications Office of the European Union, 2015, ISSN 978-92-79-51017-5 | <https://crm.jrc.ec.europa.eu/p/IRMM-427> |
| IRMM-428 | perfluoroalkyl substances in water | M. Dabrio *et al.*, The certification of the mass concentration of perfluoroalkyl substances (PFASs) in water:IRMM-428, EUR 27430, Luxembourg: Publications Office of the European Union, 2015, ISBN 978-92-79-51037-3 | <https://crm.jrc.ec.europa.eu/p/IRMM-428> |
